# Brain‐derived neurotrophic factor overexpression in taste buds diminishes chemotherapy induced taste loss

**DOI:** 10.1111/ejn.15799

**Published:** 2022-08-31

**Authors:** Irina Vukmanovic Nosrat, Jerry L. Palacios, Steven Kezian, Gloria Luong, Andrew Tran, Kim Vu, Bradley S. Henson, Philip Nosrat, Kabirullah Lutfy, Christopher A. Nosrat

**Affiliations:** ^1^ College of Dental Medicine Western University of Health Sciences Pomona California USA; ^2^ Graduate College of Biomedical Sciences Western University of Health Sciences Pomona California USA; ^3^ Department of Pharmaceutical Sciences, College of Pharmacy Western University of Health Sciences Pomona California USA

**Keywords:** brain‐derived neurotrophic factor, vismodegib, prevention, side effects, taste loss, chemotherapy

## Abstract

Vismodegib is used in patients suffering from advanced basal cell carcinoma (BCC), but 100% of the patients taking it report dysgeusia and 50% discontinue the treatment. Treatment with neurotrophic factors can stimulate neuronal survival and functional improvement in injured organs. Here, we analysed novel transgenic mouse lines in which brain‐derived neurotrophic factor (BDNF) is overexpressed in taste buds, to examine whether higher levels of BDNF would reduce or prevent negative side effects of vismodegib in the taste system. BDNF plays crucial roles for development, target innervation, and survival of gustatory neurons and taste buds. The behavioural test in this study showed that vehicle‐treated wild‐type mice prefered 10 mM sucrose over water, whereas vismodegib treatment in wild‐type mice caused total taste loss. Gustducin‐BDNF mice had a significantly increased preference for low concentration of sucrose solution over water compared to wild‐type mice, and most importantly the transgenic mice were able to detect low concentrations of sucrose following vismodegib treatment. We evaluated taste cell morphology, identity, innervation and proliferation using immunohistochemistry. All drug‐treated mice exhibited deficits, but because of a possible functional upcycled priming of the peripheral gustatory system, GB mice demonstrated better morphological preservation of the peripheral gustatory system. Our study indicates that overexpression of BDNF in taste buds plays a role in preventing degeneration of taste buds. Counteracting the negative side effects of vismodegib treatment might improve compliance and achieve better outcome in patients suffering from advanced BCC.

## Graphical abstract

A schematic module of the role of BDNF and chemotherapy induced taste lossIncreased levels of BDNF in GB mice have elevated levels of TrkB, NCAM and P2X3, which have protective properties. Upcycled priming of BDNF has therapeutic effect, can mitigate deterioration of the taste system and prevent taste loss.

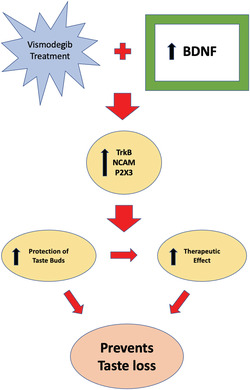

**List of Abbreviations**
BCC: basal cell carcinoma
BDNF: brain‐derived neurotrophic factor
GB: Gustducin‐BDNF
Gust‐BDNF: Gustducin‐BDNF
Hh: hedgehog
NCAM: neural cell adhesion molecule
NTRK2: neurotrophic receptor tyrosine kinase 2
P2X3: purinergic receptor P2X3
Shh: sonic hedgehog
T1R3: taste 1 receptor member 3

## 1 INTRODUCTION

Vismodegib (Erivedge®) is a hedgehog (Hh) pathway inhibitor given to patients with metastatic or with locally advanced basal cell carcinoma (BCC) (Ally et al., 2014; Sekulic et al., 2012) by binding and interfering with smoothened, and thereby suppressing an aberrant activation of the pathway. Because of vismodegib's low toxicity and specificity for the Hh pathway, this drug has advantages compared with conventional chemotherapy (De Smaele et al., 2010). However, despite vismodegib's relatively good safety and tumour shrinkage, it also causes adverse effects. Dysgeusia and muscle spasms were reported in 100% of the patients (Gonzalez et al., 2019), and approximately 50% of patients discontinue taking the drug (Sekulic et al., 2012). This is also confirmed with studies in which vismodegib‐treated mice have altered taste perception, reduced taste bud size, and decreased number of taste cells, including Phospholipase Cß2‐ and α‐Gustducin‐positive cells, which are the primary cells for detecting sweet, bitter and umami tastants (Mistretta & Kumari, 2019; Yang et al., 2014).

Brain‐derived neurotrophic factor (BDNF) is the most pivotal neurotrophic factor in the taste system. BDNF is present in taste bud progenitor cells before the arrival of nerve fibres and is required for innervation and development of taste buds. In the absence of BDNF, many taste buds are lost, gustatory papillae are severely malformed, and proper innervation and taste discrimination is lost (Cooper & Oakley, 1998; Ito & Nosrat, 2009; Nosrat et al., 1997). It continues to be expressed in adult taste buds in rodents and humans (Meng et al., 2015; Nosrat et al., 2000), indicating involvement in maintenance and regeneration of taste cells, and for neural plasticity. Similar findings have been reported in other sensory and neural systems (Canals et al., 2004; Ernfors et al., 1995; Fundin et al., 1997; Yang et al., 2011). Although many studies have shown that exogenous neurotrophic factors improve deficits, the delivery method appears to play an important role (Eriksdotter‐Jönhagen et al., 2012; Yates et al., 2004).

Our laboratory has generated novel BDNF overexpressing Gustducin‐BDNF (GB) mice with taste cell‐specific overexpression of BDNF and phosphorylated TrkB, denser gustatory and general innervation, an upregulation of BDNF, neurotrophic receptor tyrosine kinase 2 and neural cell adhesion molecule 1, which lead to an increased number of taste cells and larger taste buds (Nosrat et al., 2012). This study aimed to explore whether taste‐specific increased levels of BDNF in mutant mice has positive effects on taste perception, nerve fibres and survival of taste cells following vismodegib treatment. After confirming that overexpression of BDNF protects the morphology of taste buds and innervation, taste preference testing was analysed. Our method enables us to analyse taste dysfunction, which allows for a variety of future applications involving taste disturbances.

## 2 MATERIALS AND METHODS

### 2.1 Animals

Five‐ to seven‐month old male and female homozygous GB transgenic mouse lines and wild‐type controls obtained from Jackson Laboratories (C57BL/6J, Bar Harbour, ME, USA) were used in this study. Generation of GB mouse lines, denoted GB739 and GB759, have been reported previously, and the genetic background is C57BL/6J (Nosrat et al., 2012). The average weights of GB739 and GB759 mice were 27.0 g for the males and 19.5 g for the females. For the C57 mice, the corresponding weights were 29.7 g for the males and 21.8 g for the females. No mice lost more than 10% body weight during the study. The mice were randomly divided into groups with equal distribution of males and females, and no significant sex differences were identified. The mice were housed in groups of four to five mice per cage using individually ventilated caging system under a 12‐h light/dark cycle in a temperature‐ and humidity‐controlled environment with food and water ad libitum. All animal studies were in accordance with the National Institutes of Health and approved by the Institutional Animal Care and Use Committee at Western University of Health Sciences (Pomona, CA, USA).

### 2.2 Vismodegib administration

GB739, GB759 and wild‐type controls (*n* = 15/genotype) were administered vismodegib (LC Laboratories, Woburn, MA; 30 mg/kg) orally in almond butter (Jif® Natural Creamy Almond Butter Spread) on a glass petri dish for 10 weeks. A previous study has induced taste loss by administering daily oral gavage of 30 mg/kg vismodegib for 3.5 months (Yang et al., 2014). Because the wild‐type mice showed total taste loss succeeding 10 weeks of vismodegib treatment, we terminated the drug treatment at this point. Oral administration was chosen in order to reduce the stress and discomfort of handling of the mice, as well as to avoid any risk of injury known to be associated with oral gavage (Gonzales et al., 2014). Additionally, this mimics the appropriate administration of vismodegib in patients. On the day prior to initiation of vismodegib administration, the mice were separated from their group and placed in individual cages with one mouse per cage in order to achieve equal dosing among mice. Once all wild‐type and GB mice ingested the drug in its entirety, they were placed back into their home cages. This experimental design allowed that all mice were treated uniformly. The control group received almond butter only, which was used as vehicle for vismodegib. The mice were habituated to oral administration by smearing a thin layer of almond butter on the water bottles once a day for 7 days during acclimatisation. Vismodegib and almond butter were mixed with a spatula for 10 min on a paper pad, which was added into pellet molds (Ambrose Mesa Mold, Prod #107, Ted Pella) and stored at −20°C until use. Each mold consisted of 12 round wells, and the mice received one pellet a day containing 100 mg almond butter and .59–.89 mg vismodegib depending on the weight of the mice. Vehicle was administered to the non‐treated control groups (*n* = 10/genotype).

### 2.3 Taste preference test

Two‐bottle testing was performed by using automated contact lickometer chambers (Med‐Associates, VT, USA), housed in a temperature‐controlled holding room, which detect and record the number of licks. Each mouse was initially habituated to the lickometer chamber (Eylam & Spector, 2002), in which mice were presented for 3 days with two sipper bottles filled with distilled water in each cage. The mice had 5–10 g of food pellets a day. After the mice were acclimatised to the experimental environment, they were presented with a bottle containing 10‐mM sucrose solution and a bottle containing distilled water for 48 h (Yang et al., 2014). The licks were recorded for 11 h during the night as mice are nocturnal animals and most active during this period. The position of the bottles was switched the following day. The mean lick count over 22 h was calculated for sucrose and water. Taste preference was calculated by the ratio of tastant intake to tastant and water intake (Schroer et al., 2021). Only lick‐counts from stable recordings were used for data analysis.

### 2.4 Immunofluorescence microscopy

Following vismodegib/vehicle treatment and testing for taste preference, GB739, GB759 and wild‐type mice were euthanised by isoflurane overdose, perfused transcardially through the ascending aorta with phosphate‐buffered saline (PBS), followed by 2% paraformaldehyde in PBS. The tongues were dissected, post‐fixed for 1 h, cryoprotected with 10% sucrose and stored at 4°C until use. Circumvallate papillae, one of the gustatory papillae, were chosen for analysis because they contain over 200 taste buds in the trench wall of the paplillae (Sohn et al., 2011). Circumvallate papillae were imbedded in Tissue‐Tek® optimum cutting tissue compound; 14‐μm cryosections were generated, dried and washed 3 times for 10 min with PBS. Immunohistochemical detection of Sonic Hedgehog (Shh, rabbit, Santa Cruz, 1:100), Ki67 (rat, eBioscience, 1:100), Taste 1 receptor member 3 (T1R3, goat, Santa Cruz Biotechnology, 1:500), α‐Gustducin (rabbit, Santa‐Cruz, 1:200), Troma‐1 (rat, Hybridoma bank, 1:80), neural cell adhesion molecule (NCAM, rabbit, Chemicon, 1:400) and purinergic receptor P2X3 (P2X3, guinea pig, Chemicon, 1:500) was performed on paraformaldehyde pre‐fixed sections and incubated overnight at 4°C if nothing else was noted. Secondary antibodies were applied and incubated for 90 min at room temperature in dark using the corresponding fluorescent dye‐conjugated antibodies. All antibodies were diluted in .3% Triton‐X 100 in PBS. Slides were cover slipped with glycerol in PBS (1:2). For detection of T1R3, NCAM and P2X3, slides were incubated in blocking solution with 5% normal goat serum and 1% bovine serum albumin for 60 min prior to the incubation with the antibodies. Only GB739 or GB759 are shown to reduce redundancy. Images were acquired using Nikon 80i or Olympus Model BX40 fluorescence microscopes, and ACT‐1 software.

### 2.5 Fluorescence signal quantification

Immunofluorescence sections were examined using Nikon 80i or Olympus Model BX40 fluorescence microscopes and ACT‐1 software. Images were taken at 20X magnification. Output images were analysed using ImageJ (v.1.50; National Institutes of Health) to quantify antibody expression (Kondo et al., 2014). Control and experimental tissues from all mice were included on the same slide, in order to achieve equal exposure and fading, and thus providing a better approach for relative optical density comparison. The evaluators were blinded to the treatment, genotype and antibody during analysis of taste bud images. Five areas of taste bud sections (*n* = 5/genotype) were randomly selected in the region of interest, outlined and optical density of Shh, Ki67, α‐Gustducin, T1R3, NCAM and P2X3 was measured.

### 2.6 Circumvallate taste bud measurements

To examine the possible effect of vismodegib on the size of circumvallate papillae, 14‐μm thick sections were incubated with Troma‐1. The circumvallate papillae were photographed midway on transverse sections. Midway was defined based on the number of tissue sections containing the papillae, and the section in the middle was selected for analysis and photographed according to the methods previously described in Nosrat et al. (2004). Circumvallate taste buds were measured on the midway section of the circumvallate papillae by using ImageJ. Maximum widths for each taste bud on the sections were measured perpendicular to the long axis in wild‐type and GB mice (either GB739 or GB759) (*n* = 5/genotype).

### 2.7 Sonic hedgehog labelling in circumvallate papillae

Double immunohistochemistry (IHC) was performed with Shh and Troma‐1 on sections of wild‐type and GB (either GB739 or GB759) circumvallate papillae, which identified Shh + cells in taste buds. Circumvallate papillae sections were photographed, and Shh (red) and Troma‐1 (green) were merged in Adobe Photoshop. The “difference” function was used in order to visualise Shh + cells in the taste buds.

## 3 GENOTYPING

Tail samples were acquired from each mouse for genotyping. Tail biopsies were digested overnight at 55° with proteinase K, DNA was isolated and polymerase chain reaction (PCR) was conducted in a final reaction mixture volume of 14.6 μl including 5 μl of the isolated DNA. The primers used for GB were GB forward: 5′‐CTA CGA GAC CAA GTG TAA TCC‐3′ and GB backward: 5′‐CCC CCC AGA ATA GAA TGA C‐3′. PCR conditions were as follows: a preheating step for 3 min at 94°C followed by 35 cycles of 45 s at 94°C, 30 s at 54°C, and 45 s at 72°C, and a final extension step of 4 min at 72°C. The PCR fragments were separated according to size using electrophoresis on a 1.5% agarose gel.

### 3.1 Statistical analysis

Statistical analysis was performed using GraphPad Prism 7.0 software. Two‐way analysis of variance (ANOVA) was used to determine differences between treatment and genotype. Tukey post hoc analysis was carried out to determine the significance between the groups. The values in the text and graphs are presented as mean ± standard error of mean. The *p* values < .05 were considered statistically significant for all analysis.

## 4 RESULTS

### 4.1 Taste bud‐specific overexpression of BDNF improves taste preference and diminishes vismodegib‐related taste loss in GB mice

To investigate whether innate BDNF overexpression in taste buds is able to alleviate taste dysfunction, we first examined the taste preference levels in GB739, GB759 and wild‐type mice to assess the baseline. The 10‐mM sucrose solution is a reliable concentration to use as both wild‐type and GB mice show a detectable preference for this concentration. We tested the ability of both vismodegib‐ and vehicle‐treated mice to detect 10 mM sucrose by two‐bottle choice test, where one drinking bottle contains sucrose and the other distilled water. The preference ratio indicates which solution the animals favour. Preference is defined as a value higher than 50%, whereas avoidance is defined as a value lower than 50%, and indifference as values around 50%. During the preference testing, the sucrose solution was tested against water.

**FIGURE 1:**
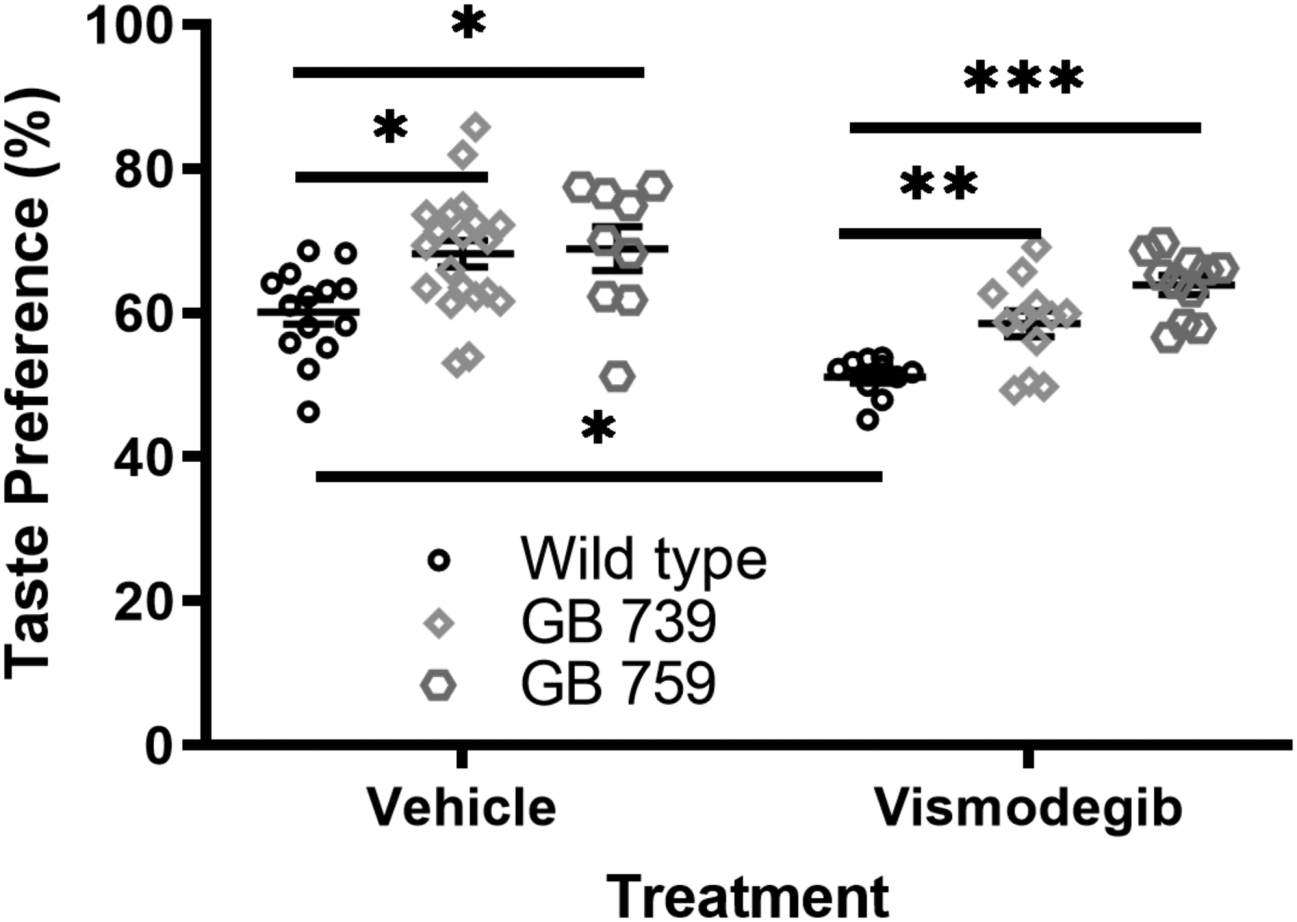
**Taste perception is preserved in BDNF overexpressing mice following vismodegib treatment.** There was a significantly higher taste preference in vehicle‐treated GB739 and GB759 transgenic mice compared to vehicle‐treated wild‐type controls. Vismodegib‐treated wild‐type mice showed a significant decrease in taste preference. Vismodegib‐treated GB759 mice presented a significantly higher preference for the sucrose solution compared to vismodegib‐treated wild‐type mice. Although there was a higher preference in vismodegib‐treated GB739 mice compared to their wild‐type mice counterparts, they did not reach the level of significance, but there is a trend. * *p* < 0.05; ** *p* < 0.01; *** *p* < 0.001

Vehicle‐treated wild‐type mice demonstrated a natural taste preference of 60.2% (± .02) for 10 mM sucrose solution. GB739 and GB759 vehicle‐treated mice had a natural preference of 68.2% (± .02) and 68.9% (± .03), respectively. Two‐way ANOVA showed that there was a significant increase in taste preference in vehicle‐treated GB739 and GB759 transgenic mice compared to wild‐type controls (*p* < .05) (Figure 1). These data suggest that BDNF overexpression not only increases taste bud size, taste cell number and innervation density (Nosrat et al., 2012) but also improves taste perception and preference for sucrose in GB mice.

We administered vismodegib daily to GB739, GB759 and wild‐type mice for 10 weeks, and investigated potential differences in their taste perception compared to the vehicle‐treated groups. We found that vismodegib treatment reduced taste preference significantly in wild‐type mice (*p* < .05). Vismodegib‐treated wild‐type mice displayed 51.1% (± .009) taste preference, which is significantly lower than the preference of vehicle‐treated wild‐type mice. Further, this indicates that the vismodegib‐treated wild‐type mice could not distinguish the sucrose solution from water. These findings suggest a loss of sweet taste sensation in wild‐type mice as a result of vismodegib treatment.

On the other hand, vismodegib‐treated GB759 mice had a preference of 63.9% (± .01), indicating that vismodegib failed to significantly reduce taste preference in these mutant mice (*p* > .05). Vismodegib‐treated GB759 mice displayed significantly higher preference for sucrose compared to the vismodegib‐treated wild‐type mice (*p* < .001). This indicates that delivery of BDNF in GB mice can prevent taste loss following vismodegib treatment. Vismodegib treatment reduced taste preference in GB739 mice (*p* < .01), but despite the decrease, GB739 mice had a taste preference of 58.5% (± .02) indicating that they were still able to discriminate between water and 10 mM sucrose solution. Vismodegib‐treated GB739 mice still had a higher preference than the vismodegib‐treated wild‐type mice. Data were analysed using two‐way ANOVA followed by the post‐hoc Tukey test. The results showed a significant effect of treatment (*F*[1,70] = 24.71; *p* < .0001) and a significant effect of genotype (*F*[2,70] = 15.25; *p* < .0001) but no significant interaction (*F*[2,70] = .7889; *p* > .05).

### 4.2 Taste bud‐specific overexpression of BDNF alleviates the decrease of taste bud size in circumvallate papillae

**FIGURE 2:**
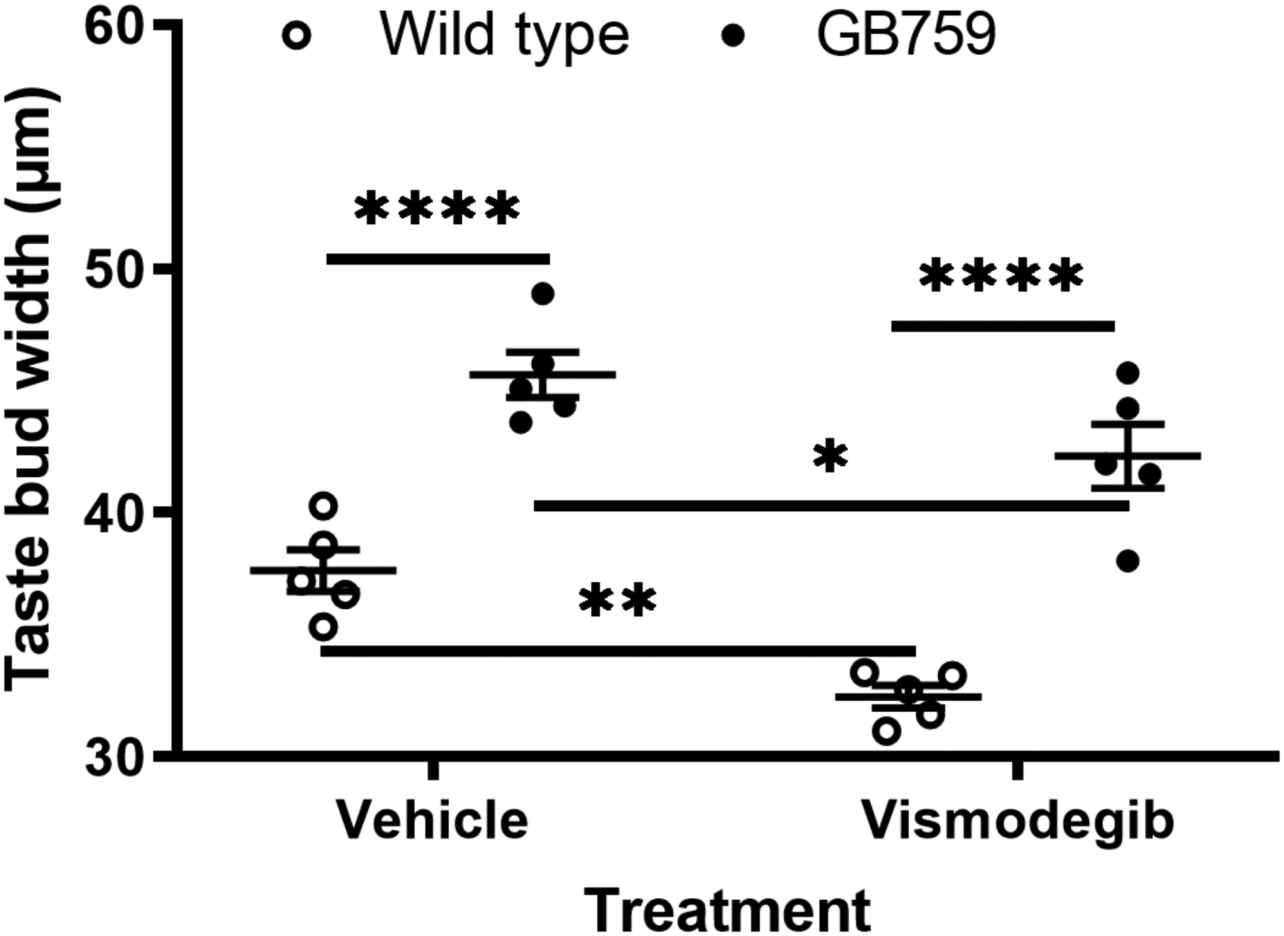
**Overexpression of BDNF in taste buds prevents the decrease of taste bud size in circumvallate papillae.** Vehicle‐treated GB mice had significantly larger taste buds than wild‐type mice. Additionally, vismodegib‐treated GB mice had larger taste buds compared to both vehicle‐ and vismodegib‐treated wild‐type mice. *p < 0.05, **p < 0.01, ****p < 0.0001

Taste bud size was quantified by measuring the width of taste buds. Troma‐1 antibody was used to visualise cytokeratin 8 in the circumvallate taste buds. We found that wild‐type mice treated with vehicle had an average taste bud width of 37.6 μm (± .9) (Figure 2). Vehicle‐treated GB mice (either GB739 or GB759) had the largest taste buds, with a width of 45.7 μm (± .9) (Figure 2), which is in agreement with our previous study (Nosrat et al., 2012). After vismodegib treatment, the taste bud width was 32.5 μm (± .5) in wild‐type mice, which represents a significant reduction (*p* = .01). Vismodegib‐treated GB mice had a taste bud width of 42.3 μm (± 1.3), showing only a small reduction in size compared to vehicle‐treated GB mice (*p* < .05); however, the decrease was modest. Most importantly, the width of vismodegib‐treated GB mice was significantly larger than the width of vehicle‐treated wild‐type mice (*p <* .001). Two‐way ANOVA revealed a significant effect of treatment ([*F*1,16] = 20.47; *p* < .001) and significant effect on genotype (*F*[1.16] = 90.93; *p* < .0001) but there was no significant interaction (*F*[1,16] = .958; *p >* .05).

### 4.3 Innervation of the peripheral gustatory system

**FIGURE 3:**
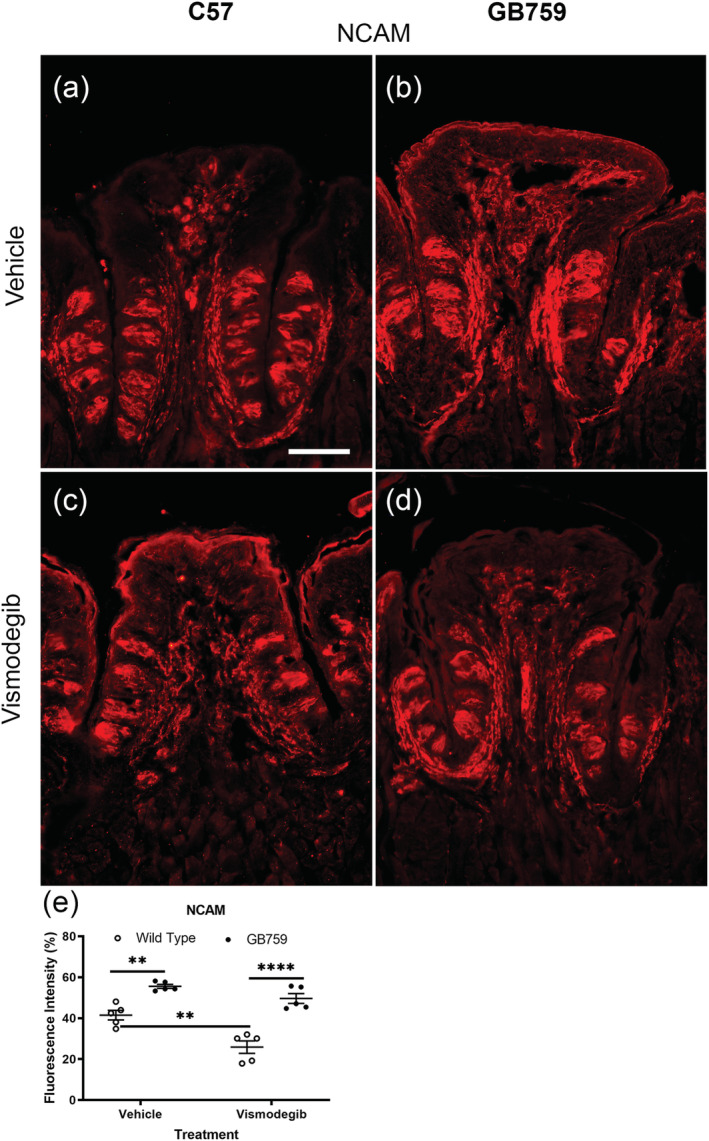
**BDNF overexpression in taste buds preserves NCAM labelling.** NCAM innervation was richer in taste buds of vehicle‐treated GB759 mice (b) compared to vehicle‐treated C57 (a) and preserved in vismodegib‐treated GB759 mice (d) compared to vismodegib‐treated C57 mice (c). The NCAM fluorescence intensity was significantly higher in both vismodegib‐ and vehicle‐treated GB759 mice compared to their wild‐type counterparts (e). There was no significant decrease in NCAM fluorescence intensity in vismodegib‐treated GB759, whereas there was a significant decrease in vismodegib‐treated wild‐type mice. Scale bar = 100 μm. **p < 0.01; **p < 0.01; ****p < 0.0001. NCAM, neural cell adhesion molecule 1

We used NCAM antibodies to analyse nerve fibres as well as label presynaptic Type III cells. We have previously demonstrated that overexpression of BDNF increases NCAM labelling and the number of Type III cells (Nosrat et al., 2012). In this experiment, we found that NCAM labelling was highest in the GB mice showing very intense staining in the taste cells suggesting an increased number of Type III taste cells. In addition, there was a high level of NCAM labelling throughout the subepithelial nerve plexus (Figure 3b). There was a reduction in NCAM labelling in all vismodegib‐treated groups (Figure 3c and d), but vismodegib‐treated GB mice had still stronger NCAM staining both in the taste buds and subepithelial nerve plexus (Figure 3d) compared to the vismodegib‐treated wild‐type mice (Figure 3c). This indicates that there were more nerve fibres and Type III cells in GB vismodegib‐treated mice compared to vismodegib‐treated wild‐type mice. The mean immunoexpression intensity of NCAM measured by ImageJ was 42% for C57 vehicle‐, 26% for C57 vismodegib‐, 56% for GB759 vehicle‐ and 50% for GB759 vismodegib‐treated mice (Figure 3e). NCAM labelling was significantly higher in vismodegib‐treated GB759 mice compared to vismodegib‐treated wild‐type mice (*p* < .0001) and was significantly decreased in vismodegib‐treated wild‐type mice compared to vehicle‐treated wild‐type mice (*p* = .001). Interestingly, there was no significant decrease between vismodegib‐treated GB mice and their vehicle control (ns). Two‐way ANOVA showed a significant effect of treatment (*F*[1,16] = 21.93; *p* < .01) and a significant effect of genotype ((*F*[1,16] = 67.67; *p* < .0001), and the interaction was (*F*[1,16] = 4.472; *p* = .05)).

**FIGURE 4:**
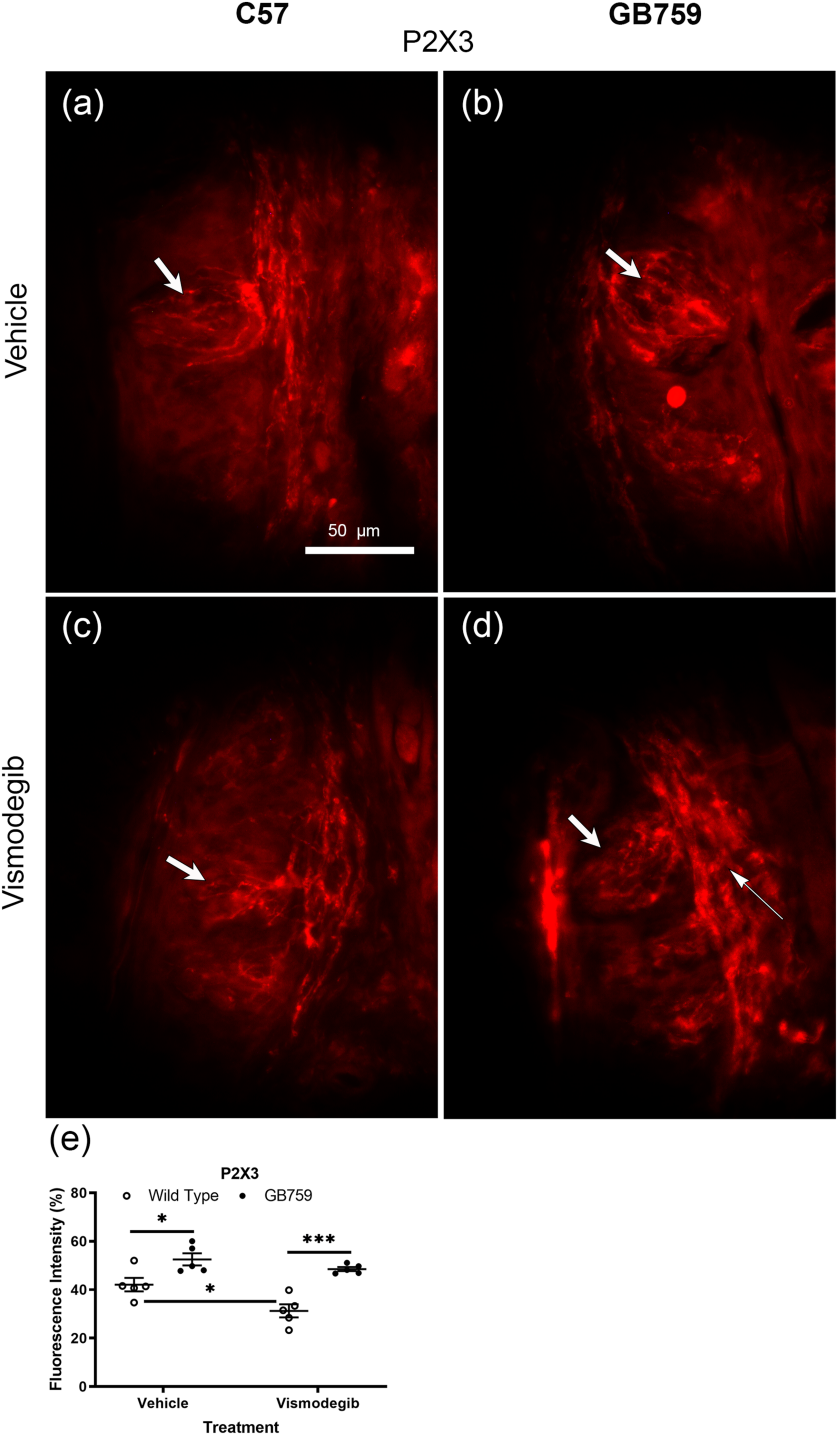
**BDNF overexpression protects P2X3 labelling of taste buds and gustatory innervation.** IHC with P2X3 antibodies showed preserved innervation inside the taste buds (d, thick arrow) and in the subepithelial nerve plexus of the circumvallate papillae of vismodegib‐treated GB759 (d, thin arrow) compared to vismodegib‐treated wild‐type mice (c). Both vismodegib‐treated (d) and vehicle‐treated (b) GB759 mice showed stronger labeling compared to their wild‐type controls (a,c) Because of the orientation of the section, the subepithelial nerve plexus is not visible in vehicle‐treated GB759mice (b). There was a significant decrease in P2X3 fluorescence intensity in vismodegib‐treated wild‐type mice (e), whereas there was no significant decrease in vismodegib‐treated GB759 mice. Scale bar = 50 µm. *p < 0.05; ***p < 0.001. IHC, immunohistochemistry

To establish whether BDNF overexpression would counteract deficits in the gustatory innervation of circumvallate taste buds, P2X3 antibodies were used to visualise the purinergic innervation. ATP is released when sweet, umami and bitter compounds interact with receptor cells, which among others bind to ATP P2X3 receptors expressed on gustatory nerve fibres (Huang et al., 2011). We observed a decrease in the P2X3 labelling in all groups receiving vismodegib (Figure 4c and d). Similar to NCAM, vismodegib‐treated GB759 had stronger labelling of P2X3 receptors and positive nerve fibres (Figure 4d) compared to vismodegib‐treated wild‐type mice (Figure 4c). P2X3 nerve fibres were denser in vehicle‐treated GB759 taste buds (Figure 4b, thick arrow) compared to those in wild‐type mice (Figure 4a, thick arrow). Vismodegib‐treated wild‐type mice showed a larger decrease in P2X3 + nerve fibres (Figure 4c, thick arrow) in the taste buds compared to GB mice (Figure 4d, thick arrow). There was a stronger labelling of P2X3 + nerve fibres in the subepithelial nerve plexus in vismodegib‐treated GB mice (Figure 4d, thin arrow) compared to wild‐type mice (Figure 4c). The mean immunoexpression intensity of P2X3 measured by ImageJ was 42% for wild‐type vehicle‐, 31% for wild‐type vismodegib‐, 53% for GB759 vehicle‐ and 49% for GB759 vismodegib‐treated mice (Figure 4e). There was no significant decrease in P2X3 fluorescence intensity in vismodegib‐treated GB759 mice compared to vehicle‐treated GB759 mice (ns), whereas there was a significant decrease in vismodegib‐treated wild‐type mice compared to the vehicle‐treated wild‐type mice (*p* < .05). The mean optical density in vismodegib‐treated GB759 mice was significantly higher than in vismodegib‐treated wild‐type mice (*p* < .001). The optical density was also higher in vehicle‐treated GB759 mice compared to vehicle‐treated wild‐type mice (*p* < .05). Two‐way ANOVA showed a significant effect of treatment (*F*[1,16] = 9.867; *p* < .01) and a significant effect of genotype (*F*[1,16] = 34.4; *p* < .0001) but no significant interaction (*F*[1,16] = 2.073; *p* > .05).

### 4.4 Immunohistochemical analysis of taste cells, taste‐modulating factors and taste progenitor cells

**FIGURE 5:**
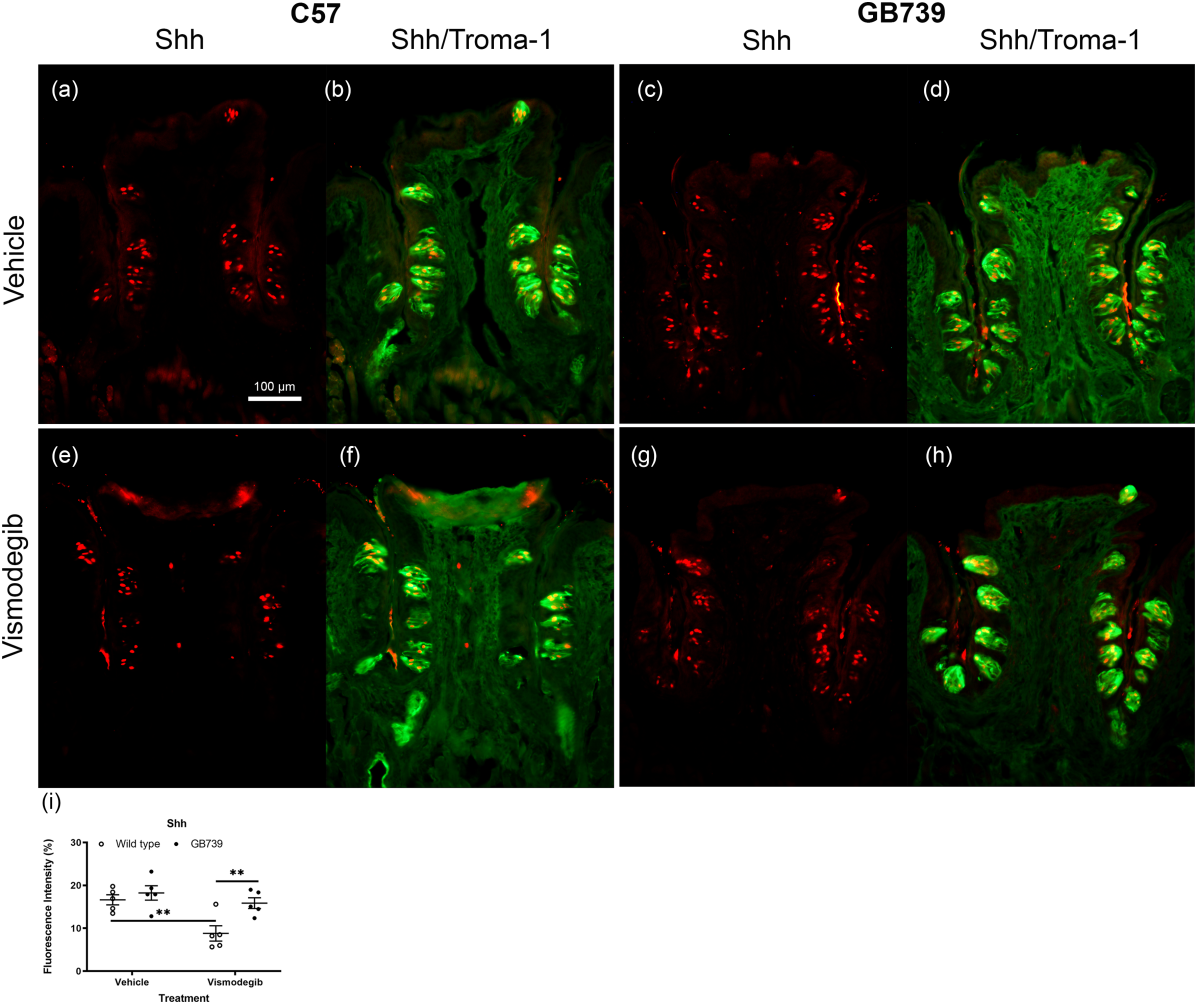
**Taste bud‐specific overexpression of BDNF counteracts the decrease of Shh‐like immunoreactive cells.** Shh and Troma‐1 labelling were examined using IHC in C57 and GB circumvallate papillae sections. Superimposition of Shh (red) and Troma‐1 (green) resulted in yellow (co‐localisation). (a, c, e and g) IHC with Shh, and (b, d, f and h) double staining with Shh and Troma‐1. (c and d) Shh and Troma‐1 expressions are highest in vehicle‐treated GB739. (e and f) Shh and Troma‐1 labelling is lower in vismodegib‐treated C57 mice than in (g and h) vismodegib‐treated GB739 mice. (i) Shh fluorescence intensity was significantly higher in vismodegib‐treated GB739 mice compared to vismodegib‐treated wild‐type mice. Vismodegib‐treated wild‐type mice showed a significantly lower Shh fluorescence intensity than vehicle‐treated wild‐type mice. Scale bar = 100 μm. *p < 0.05; **p < 0.01. IHC, immunohistochemistry; GB, Gustducin‐BDNF; BDNF, brain‐derived neurotrophic factor

Shh labelling was examined in the taste buds in vismodegib/vehicle‐treated GB739 and wild‐type mice. Double IHC with Shh and Troma‐1 antibodies was performed to visualise the Shh + taste cells, and the overlap resulted in yellow stain. Our data showed that Shh (red) was expressed in Troma‐1 + (green) taste buds in wild‐type and transgenic mice (Figure 5a–h). Shh labelling was highest in the vehicle‐treated GB739 compared to all the groups, including the vehicle‐treated wild‐type mice. There was a weaker Shh labelling in wild‐type mice following vismodegib treatment (Figure 5e and f), whereas the decrease was modest in GB739 mice (Figure 5g and h). The Shh distribution extended throughout the taste buds, which could be because of the advanced age of the mice, as they were 7.5–9.5 months old at the time of tissue collection. A study has indeed shown that Shh‐positive cells are expressed within the center of the buds in adult mice (Liu et al., 2013). The mean immunoexpression intensity (measured as optical density) of Shh measured by ImageJ was 16.6% for wild‐type vehicle‐, 8.8% for wild‐type vismodegib‐, 18.2% for GB739 vehicle‐ and 15.9% for GB739 vismodegib‐treated mice (Figure 5i). The mean Shh labelling in vismodegib‐treated wild‐type mice decreased significantly (*p* < .01), whereas the decrease in vismodegib‐treated GB739 mice was not significant (ns). Vismodegib‐treated GB mice showed a significantly higher Shh fluorescence compared to vismodegib‐treated wild‐type mice (*p* < .05). Two‐way ANOVA revealed a significant effect of treatment (*F*[1,16] = 11.71; *p* < .01) and a significant effect on genotype (*F*[1.16] = 8.407; *p* < .05) but no significant interaction (*F*[1,16] = 3.347; *p* > .05).

**FIGURE 6:**
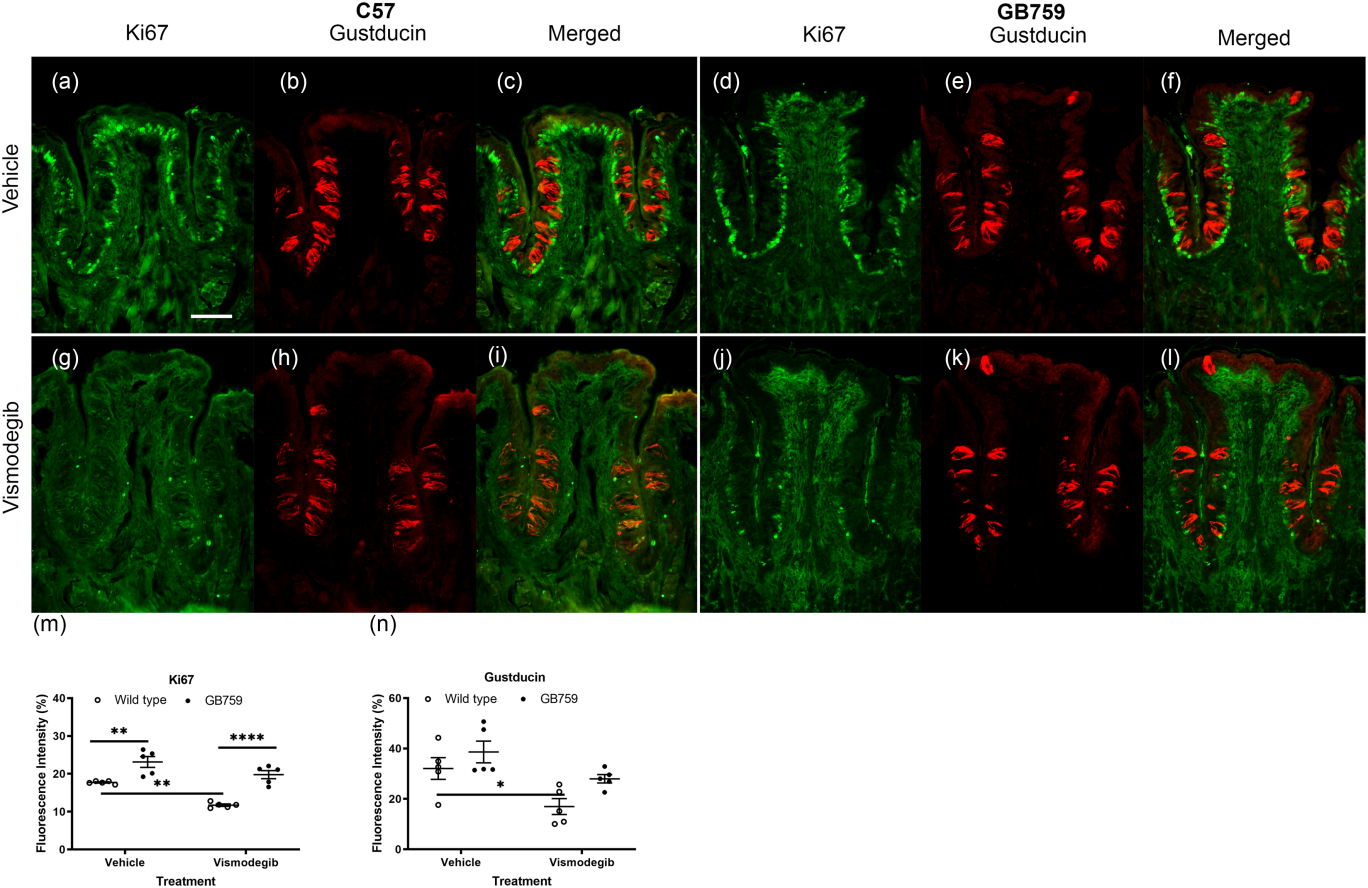
**Reduced Ki‐67 and Gustducin labelling in wild‐type vallate taste cells compared to GB mice.** (a, d, g and j) Double IHC was performed with Ki‐67 and (b, e, h and k) with Gustducin (c, f, i and l). (d) Ki67 (green) showed stronger labelling in GB759 vehicle‐treated mice compared to C57 vehicle‐treated mice (a). (g) Vismodegib‐treated C57 mice had a robust decrease in Ki67 labelling, whereas the decrease was less pronounced in GB759 drug‐treated mice (j). The same trend was observed with Gustducin (red). (e) Vehicle‐treated GB759 mice had a stronger Gustducin labelling compared to vehicle‐treated C57 mice (b). Gustducin labelling in vismodegib‐treated wild‐type mice (h) was weaker compared to vismodegib‐treated GB759 mice (k). (m and n) There was no significant Ki67, and Gustducin fluorescence intensity decrease in vismodegib‐treated GB759 mice, whereas vismodegib‐treated wild‐type mice showed a significant decrease. Scale bar = 100 μm. * *p* < 0.05; ** *p* < 0.01; **** *p* < 0.0001. IHC, immunohistochemistry; GB, Gustducin‐BDNF; BDNF, brain‐derived neurotrophic factor

To analyse the effect of vismodegib treatment and the role of Shh on taste bud progenitor proliferation rate, Ki67 antibody (green) was used to detect proliferating cells in the basal regions surrounding circumvallate taste buds (Hirota et al., 2001). Although still under investigation, it is proposed that taste cells originate from a population of progenitor cells located outside the taste buds where they proliferate (Okubo et al., 2009). As previously reported (Yang et al., 2014), taste cell proliferation rate was decreased in progenitor cells in vismodegib‐treated mice. Vehicle‐treated GB759 had the strongest Ki67 labelling (Figure 6d–f), which was only slightly decreased after 10 weeks of vismodegib treatment (Figure 6j–l). In contrast, Ki67 labelling was much weaker in vismodegib‐treated wild‐type mice (Figure 6g–i). Hence, the decrease was less pronounced in GB mice compared to wild‐type mice. The mean immunofluorescence intensity of Ki67 measured by ImageJ was 17.7% for wild‐type vehicle‐, 11.7% for wild‐type vismodegib‐, 23.2% for GB759 vehicle‐ and 19.8% for GB759 vismodegib‐treated mice (Figure 6m). The Ki67 fluorescence intensity was significantly higher in vehicle‐treated GB759 mice compared to their wild‐type counterparts (*p* < .01). The Ki67 fluorescence intensity was significantly decreased in vismodegib‐treated wild‐type mice compared to the vehicle‐treated wild‐type mice (*p* < .01). The intensity was significantly higher in vismodegib‐treated GB759 mutant mice compared to vismodegib‐treated wild‐type mice (*p* < .001). Furthermore, Ki67 fluorescence was only slightly decreased in vismodegib‐treated GB759 mice compared to the vehicle‐treated mutant mice (*p* > .05, ns). Two‐way ANOVA revealed a significant effect of treatment (*F*[1,16] = 25.89; *p* = .001) and a significant effect on genotype (*F*[1.16] = 53.77; *p* < .001) but no significant interaction (*F*[1,16] = 2.063; *p* > .05).

**FIGURE 7:**
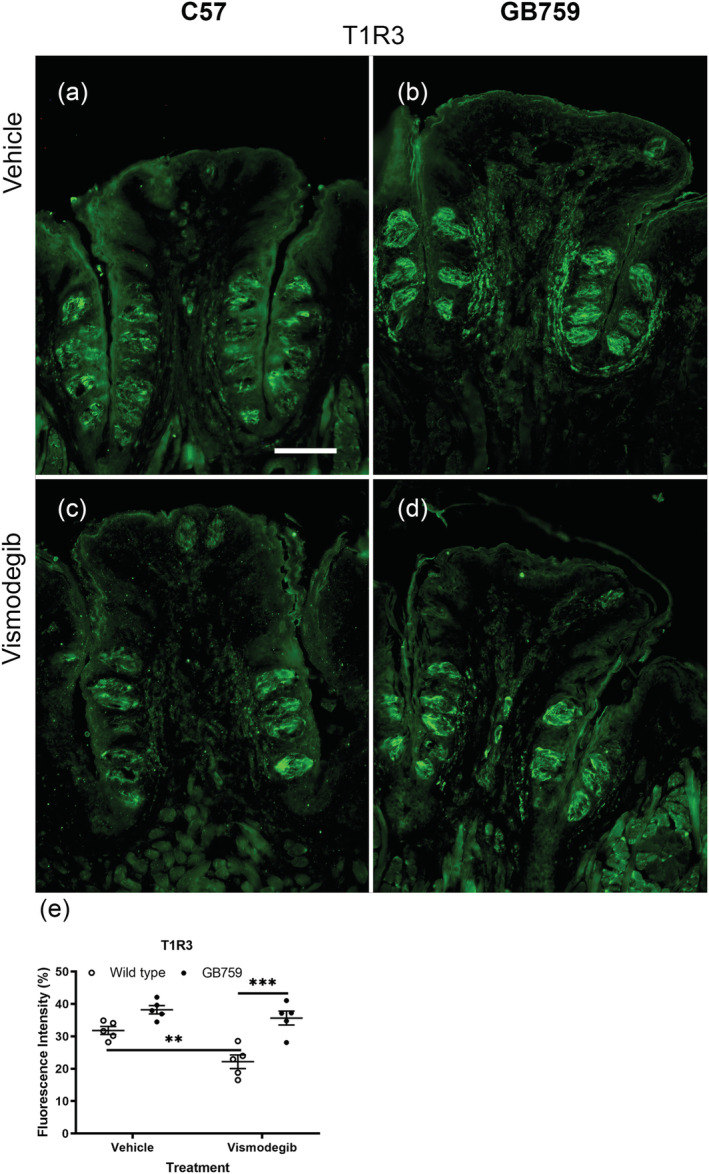
**Preserved T1R3 labelling in vismodegib‐treated GB mice compared to wild‐type mice.** IHC of circumvallate papillae showed stronger T1R3 labelling in vehicle‐treated GB circumvallate taste buds (b) compared to wild‐type mice (a). Vismodegib‐treated GB759 showed less decrease of T1R3 signals (d) compared to wild‐type vismodegib‐treated mice (c). (e) There was no significant decrease in T1R3 fluorescence intensity in vismodegib‐treated GB759 mice, whereas there was a significant decrease in vismodegib‐treated wild‐type mice. The fluorescence intensity in vismodegib‐treated GB759 mice was significantly higher compared to vismodegib‐treated wild‐type mice. Scale bar = 100 μm. **p < 0.01; ***p < 0.0001. IHC, immunohistochemistry; GB, Gustducin‐BDNF; BDNF, brain‐derived neurotrophic factor

T1R3 is a prerequisite for sweet taste detection and is expressed in Type II taste cells. In light of taste deficits in wild‐type mice after vismodegib treatment, we examined possible alterations to T1R3 labelling (Figure 7d). T1R3 labelling was weaker following vismodegib treatment in all groups, but vismodegib‐treated GB759 (Figure 7d) showed a stronger labelling compared to the vismodegib‐treated wild‐type mice (Figure 7c). The mean optical density of T1R3 measured by ImageJ was 31.8% for C57 vehicle‐, 22.17% for C57 vismodegib‐, 38.2% for GB759 vehicle‐ and 35.7% for GB759 vismodegib‐treated mice (Figure 7e). T1R3 labelling decreased significantly in vismodegib‐treated wild‐type mice compared to vehicle‐treated wild‐type mice (*p* < .05). The T1R3 optical density in vismodegib‐treated GB759 mice decreased only slightly and was not significant (ns). The fluorescence intensity in vismodegib‐treated GB759 mice was significantly higher compared to vismodegib‐treated wild‐type mice (*p* < .001). Two‐way ANOVA showed a significant effect of treatment (*F*[1,16] = 12.27; *p* < .01) and a significant effect of genotype (*F*[1,16] = 32.6; *p* < .0001). The interaction (*F*[1,16] = 4.118; *p* = .059) almost reached significance.

To confirm the results of decreased expression of T1R3, taste cells were labelled with α‐Gustducin, which is a subunit of a heterotrimeric protein. T1R3 is a G protein‐coupled receptor, which upon binding of sweet or umami tastants interacts with α‐Gustducin. The trend for α‐Gustducin was the same as for T1R3. A decrease of labelling was observed in both wild‐type (Figure 6h) and GB (Figure 6k) vismodegib‐treated mice, but the reduction was less in the BDNF overexpressing mouse line (Figure 6k). The mean immunoexpression intensity of α‐Gustducin measured by ImageJ was 32% for wild‐type vehicle‐, 17% for wild‐type vismodegib‐, 39% for GB759 vehicle‐ and 28% for GB759 vismodegib‐treated mice (Figure 6n). There was a significant decrease in vismodegib‐treated wild‐type mice compared to the vehicle‐treated wild‐type mice (*p* < .05). There was no significant decrease in vismodegib‐treated GB759 mice compared to vehicle‐treated GB759 mice (*p* > .05). Two‐way ANOVA revealed a significant effect of treatment (*F*[1,16] = 13.37; *p* < .01) but no significant effect on genotype (*F*[1.16] = 6.24; *p* = .02) and no significant interaction (*p* > .05).

## 5 DISCUSSION

The current study is the first to show that site‐specific overexpression of BDNF in taste buds diminishes vismodegib‐induced taste loss and partially protects taste bud morphology. We utilised a transgenic mouse model generated in C57BL/6J (taster) background, in which BDNF overexpression is driven by a well‐characterised promoter for α‐Gustducin, causing significant increased levels of different molecules, but most importantly upregulated levels of BDNF mRNA and protein in lingual taste buds (Nosrat et al., 2012). In the present study, we demonstrate that vehicle‐treated GB mice exhibit a significant increase in preference for very low concentration of sucrose solution compared to vehicle‐treated wild‐type mice. This is indeed a significant finding in itself, which is extending our finding of our previous study (Nosrat et al., 2012). Despite the decrease in taste preference in the GB mice following vismodegib treatment, the mutant mice are still able to distinguish the low concentration of 10‐mM sucrose over water, whereas the wild‐type mice experienced a loss of preference following vismodegib treatment. We propose that the low concentration of sucrose in our study clearly distinguishes taste function rather than caloric intake or hedonic phase of sucrose ingestion.

Vismodegib‐treated GB mice exhibit larger taste buds than vismodegib‐treated wild‐type mice. This might indicate an interaction between BDNF and Shh signalling, and possible amplification of Shh signalling. BDNF and Shh, both involved in development, are crucial for synaptic plasticity and neurogenesis in many nervous system structures (Friedman et al., 1991; Lee & Son, 2009; Lindholm et al., 1997; Yao et al., 2016). Shh is expressed in post‐mitotic precursor cells (Type IV or basal cells) in taste buds, plays an important role in proliferation of progenitor cells (Liu et al., 2013; Miura et al., 2006) and is associated with taste bud maintenance (Liu et al., 2013; Miura et al., 2001). It is interesting to note that Shh is expressed in gustatory neurons, indicating nerve‐derived Shh may maintain taste buds (Castillo‐Azofeifa et al., 2017), and it has been shown that vismodegib inhibits the pathway (De Smaele et al., 2010), causing a decreased number of Shh + taste cells (Yang et al., 2014), which is in alignment with our findings. The Hh pathway plays an essential role in organogenesis during development and plays critical roles in adult tissue maintenance, renewal and repair. Shh signalling is initiated by Shh ligand binding to the receptor patched 1. Mutation of *PATCHED 1* relieves the inhibition of smoothened and causes an activated Hh pathway leading to uncontrolled proliferation resulting in BCC (Epstein, 2008; Gailani & Bale, 1997). Taste buds are sensory end organs, consisting of 50–100 Type I, Type II, Type III and a small number of Type IV cells. They perceive the five basic tastants, which are sweet, salt, sour, bitter and umami (Chaudhari & Roper, 2010), as well as the taste for carbonation (Chandrashekar et al., 2009; Sternini, 2013) and possibly fat (Camandola & Mattson, 2017). Basal cells are the Shh expressing cells and have been proposed to differentiate into the other cell types in taste buds (Miura et al., 2014). Because the Shh labelling is decreased in vismodegib treated mice, a lower number of basal cells can differentiate into mature cells, leading to decreased taste preference which is most affected in wild‐type mice. Ki67 is a cell proliferation marker expressed in all active stages of the cell cycle (Gerdes et al., 1983). Significantly better preserved Ki67 labelling in gustatory epithelia of GB mice might be because of an internal, taste bud‐specific, BDNF/TrkB activation and signalling for maintaining functional peripheral taste system, despite inhibition of Shh signalling in vismodegib‐treated GB mice. It has been posited that taste cells originate directly from perigemmal cells located immediately outside the taste buds in the oral epithelium and need to be renewed continuously as they have a limited lifespan (Okubo et al., 2009).

T1R3‐ and α‐Gustducin‐like immunoreactivity in Type II cells are stronger in the GB mice following vismodegib treatment compared to the wild‐type mice. T1R3, which is expressed on Type II receptor cells, is a subunit of the heterodimers T1R2/T1R3 and T1R1/T1R3, and mediates detection of sweet and umami tastants, respectively (Nelson et al., 2001). Upon binding, these G protein‐coupled receptors interact with α‐Gustducin, an important downstream player in taste transduction (McLaughlin et al., 1992). We propose that higher numbers of Shh + cells, which lead to higher numbers of proliferating (Ki67+) and well‐differentiated Type II cells in taste buds of GB mice, might be involved in preserving sweet taste detection in these mice compared to wild‐type mice. Additionally, the GB mice have upregulated levels of T1R3 mRNA as a result of BDNF overexpression (Nosrat et al., 2012).

Studies have shown that BDNF has neuroprotective properties and has growth‐promoting effects on injured organs, which are in particular attributed to the downstream effects of TrkB receptor signalling (Keefe et al., 2017). Both BDNF and TrkB (phosphorylated and non‐phosphorylated) are enhanced in the GB mice (Nosrat et al., 2012). Studies of BDNF and TrkB transgenic mice have proven that BDNF is required for the survival of taste cells and for preserving taste buds (Cooper & Oakley, 1998; Fritzsch et al., 1997; Meng et al., 2015; Nosrat et al., 1997), clearly indicating that BDNF binding to TrkB, in either autocrine and/or paracrine manner in taste buds, followed by phosphorylation of TrkB and activation of their downstream signalling elements are all important steps in promoting survival and renewal of taste cells. Our study shows that BDNF overexpression stabilises taste bud size after vismodegib treatment illustrated by Troma‐1 antibodies, which label cytokeratin 8 (Knapp et al., 1995; Zhang et al., 1995). We propose that the preservation of taste bud size in vismodegib‐treated GB mice is because of the effects of BDNF on synaptic plasticity and/or BDNF‐dependent promotion of taste cell survival. The GB mice have an increased number of taste cells, both in circumvallate and fungiform papillae, and have larger taste buds compared to wild‐type mice (Nosrat et al., 2012), which obviously provides them with a better baseline before the vismodegib treatment, thus possesses the priming effect indicating that overexpression of BDNF in taste buds has a functional significance.

As target‐derived neurotrophic factors regulate the neuronal survival (Davies et al., 1986), we analysed the innervation pattern with multiple nerve markers. GB mice exhibit larger taste buds, and taste bud‐specific BDNF levels are increased. The findings of the present study showed that vismodegib‐treated GB mice preserve NCAM+ nerve fibres and Type III (NCAM+) taste cells. NCAM is important for synaptogenesis and is used to label nerve fibres in the subepithelial nerve plexus in gustatory papillae, as well as to identify Type III taste cells (Nelson & Finger, 1993; Takeda et al., 1992), and NCAM levels (both mRNA and protein) are elevated in the taste buds of GB mice (Nosrat et al., 2012). Studies have shown that BDNF stimulates NCAM expression (Vutskits et al., 2001), and TrkB binding to NCAM stimulates phosphorylation of NCAM‐dependent neurite outgrowth (Cassens et al., 2010). This suggests that increased BDNF and TrkB levels in GB mice act positively on neurogenesis, synaptogenesis, and synaptic plasticity. We have previously shown that the GB mice have a denser P2X3 innervation (Nosrat et al., 2012). Gene‐knockout experiments showed that P2X2/P2X3‐deficient mice have almost no responses to several taste stimuli and severe loss of neural responses (Finger et al., 2005). In accordance with this, we showed that the GB mice indeed have denser P2X3 gustatory innervation than wild‐type mice after vismodegib treatment.

In this study, vismodegib was administered orally in almond butter mixture in order to mimic a normal patient scenario and how they would take the medication. The mice were 5–7 months old at the beginning of the drug treatment, which corresponds to a human age of about 30 to 42 years (http://www.age-converter.com/mouse-age-calculator.html). BCC prevalence increases with age (Cox, 1992), and vismodegib treatment causes dysgeusia in all BCC patients (Ally et al., 2014). Thus, our study indicates that increased levels of BDNF might be a good supplement to vismodegib treatment in order to reduce taste dysfunction in plausible age‐matched human patients receiving this drug. Because half of BCC patients discontinue taking vismodegib (Sekulic et al., 2012), counteracting adverse events might improve compliance in achieving better patient outcome in regard to cancer treatment. At the time we started this study, vismodegib was the only Shh inhibitor approved by the Food and Drug Administration, which was the reason why this drug was chosen. Today sonidegib is also approved for treatment of advanced BCC (Casey et al., 2017). Other methods to inhibit Hh pathway have also been used. Shh has been ablated in transgenic mice in which conditional neuronal driver, Thy1^CreER^, has been employed (Lu et al., 2018).

Even though wild‐type mice were not able to distinguish the low concentraton of 10‐mM sucrose. The taste bud morphology has not been severely affected in this study, which might be because of fast clearance rate of vismodegib (Dutta et al., 2017). A study has shown that TRCs undergo normal regeneration following ablation by Hh pathway antagonist treatment, attributing Shh expression in distant sensory nerves to paly an important role in this process (Lu et al., 2018). We propose that the morphology of the vismodegib‐treated mice has been partially rescued by normal regeneration. During the 10‐week long vismodegib treatment, there seems to have occurred normal regeneration counteracting the adverse events on taste buds of vismodegib. We believe that this is the reason why taste buds in vismodegib‐treated mice did not undergo larger decrease in size and signalling molecules.

To our knowledge, this is the first study to demonstrate that adverse effects of vismodegib can be mitigated. The results of this study show that innate overexpression of BDNF in taste buds reduces taste dysfunction, preserves the morphology of the peripheral taste system and protects innervation following vismodegib treatment. These results show the importance of BDNF in the taste buds and how its overexpression can attenuate the negative effects of vismodegib treatment on the peripheral taste system. It is plausible that co‐delivery of BDNF and vismodegib might be beneficial in therapy regimens for patients suffering from BCC.

## 6 CONCLUSION

Our data demonstrate that vismodegib‐induced taste loss can be prevented. We show that BDNF provides a priming effect to prevent extensive deficits in the vismodegib impaired peripheral taste system (Graphical abstract). Based on our findings, we propose that BDNF or its downstream mediators can reduce taste loss and dysfunction either prior to the start of vismodegib treatment or concurrently during treatment. This is important to address as all patients report taste loss and often discontinue taking vismodegib, in part because of impaired sense of taste.

## CONFLICTS OF INTEREST

There are no conflicts of interest.

### PEER REVIEW

The peer review history for this article is available at https://publons.com/publon/10.1111/ejn.15799.

## Data Availability

Raw data will be provided, as and when required.

## References

[ejn15799-bib-0001] Ally, M. S. , Aasi, S. , Wysong, A. , Teng, C. , Anderson, E. , Bailey‐Healy, I. , Oro, A. , Kim, J. , Chang, A. L. , & Tang, J. Y. (2014). An investigator‐initiated open‐label clinical trial of vismodegib as a neoadjuvant to surgery for high‐risk basal cell carcinoma. Journal of the American Academy of Dermatology, 71, 904–911.e901. 10.1016/j.jaad.2014.05.020 24929884

[ejn15799-bib-0002] Camandola, S. , & Mattson, M. P. (2017). Toll‐like receptor 4 mediates fat, sugar, and umami taste preference and food intake and body weight regulation. Obesity (Silver Spring, md), 25, 1237–1245. 10.1002/oby.21871 28500692 PMC5487280

[ejn15799-bib-0003] Canals, J. M. , Pineda, J. R. , Torres‐Peraza, J. F. , Bosch, M. , Martín‐Ibañez, R. , Muñoz, M. T. , Mengod, G. , Ernfors, P. , & Alberch, J. (2004). Brain‐derived neurotrophic factor regulates the onset and severity of motor dysfunction associated with enkephalinergic neuronal degeneration in Huntington's disease. The Journal of Neuroscience : The Official Journal of the Society for Neuroscience, 24, 7727–7739. 10.1523/JNEUROSCI.1197-04.2004 15342740 PMC6729627

[ejn15799-bib-0004] Casey, D. , Demko, S. , Shord, S. , Zhao, H. , Chen, H. , He, K. , Putman, A. , Helms, W. , Keegan, P. , & Pazdur, R. (2017). FDA approval summary: Sonidegib for locally advanced basal cell carcinoma. Clinical cancer Research: An Official Journal of the American Association for Cancer Research, 23, 2377–2381. 10.1158/1078-0432.CCR-16-2051 28073840

[ejn15799-bib-0005] Cassens, C. , Kleene, R. , Xiao, M. F. , Friedrich, C. , Dityateva, G. , Schafer‐Nielsen, C. , & Schachner, M. (2010). Binding of the receptor tyrosine kinase TrkB to the neural cell adhesion molecule (NCAM) regulates phosphorylation of NCAM and NCAM‐dependent neurite outgrowth. The Journal of Biological Chemistry, 285, 28959–28967. 10.1074/jbc.M110.114835 20605779 PMC2937923

[ejn15799-bib-0006] Castillo‐Azofeifa, D. , Losacco, J. T. , Salcedo, E. , Golden, E. J. , Finger, T. E. , & Barlow, L. A. (2017). Sonic hedgehog from both nerves and epithelium is a key trophic factor for taste bud maintenance. Development, 144, 3054–3065. 10.1242/dev.150342 28743797 PMC5611957

[ejn15799-bib-0007] Chandrashekar, J. , Yarmolinsky, D. , von Buchholtz, L. , Oka, Y. , Sly, W. , Ryba, N. J. , & Zuker, C. S. (2009). The taste of carbonation. Science (New York, N.Y.), 326, 443–445. 10.1126/science.1174601 19833970 PMC3654389

[ejn15799-bib-0008] Chaudhari, N. , & Roper, S. D. (2010). The cell biology of taste. The Journal of Cell Biology, 190, 285–296. 10.1083/jcb.201003144 20696704 PMC2922655

[ejn15799-bib-0009] Cooper, D. , & Oakley, B. (1998). Functional redundancy and gustatory development in BDNF null mutant mice. Brain Research Developmental Brain Research, 105, 79–84. 10.1016/S0165-3806(97)00167-3 9473598

[ejn15799-bib-0010] Cox, N. H. (1992). Basal cell carcinoma in young adults. The British Journal of Dermatology, 127, 26–29. 10.1111/j.1365-2133.1992.tb14820.x 1637690

[ejn15799-bib-0011] Davies, A. M. , Thoenen, H. , & Barde, Y. A. (1986). Different factors from the central nervous system and periphery regulate the survival of sensory neurones. Nature, 319, 497–499. 10.1038/319497a0 3945332

[ejn15799-bib-0012] De Smaele, E. , Ferretti, E. , & Gulino, A. (2010). Vismodegib, a small‐molecule inhibitor of the hedgehog pathway for the treatment of advanced cancers. Current opinion in investigational drugs (London, England: 2000), 11, 707–718. 20496266

[ejn15799-bib-0013] Dutta, R. , Kumar, V. , Peng, Y. , Evande, R. E. , Grem, J. L. , & Mahato, R. I. (2017). Pharmacokinetics and biodistribution of GDC‐0449 loaded micelles in Normal and liver fibrotic mice. Pharmaceutical Research, 34, 564–578. 10.1007/s11095-016-2081-3 27995525

[ejn15799-bib-0014] Epstein, E. H. (2008). Basal cell carcinomas: Attack of the hedgehog. Nature Reviews Cancer, 8, 743–754. 10.1038/nrc2503 18813320 PMC4457317

[ejn15799-bib-0015] Eriksdotter‐Jönhagen, M. , Linderoth, B. , Lind, G. , Aladellie, L. , Almkvist, O. , Andreasen, N. , Blennow, K. , Bogdanovic, N. , Jelic, V. , Kadir, A. , Nordberg, A. , Sundström, E. , Wahlund, L. O. , Wall, A. , Wiberg, M. , Winblad, B. , Seiger, Å. , Almqvist, P. , & Wahlberg, L. (2012). Encapsulated cell biodelivery of nerve growth factor to the basal forebrain in patients with Alzheimer's disease. Dementia and Geriatric Cognitive Disorders, 33, 18–28. 10.1159/000336051 22377499

[ejn15799-bib-0016] Ernfors, P. , Kucera, J. , Lee, K. F. , Loring, J. , & Jaenisch, R. (1995). Studies on the physiological role of brain‐derived neurotrophic factor and neurotrophin‐3 in knockout mice. The International Journal of Developmental Biology, 39, 799–807. 8645564

[ejn15799-bib-0017] Eylam, S. , & Spector, A. C. (2002). The effect of amiloride on operantly conditioned performance in an NaCl taste detection task and NaCl preference in C57BL/6J mice. Behavioral Neuroscience, 116, 149–159. 10.1037/0735-7044.116.1.149 11895177

[ejn15799-bib-0018] Finger, T. E. , Danilova, V. , Barrows, J. , Bartel, D. L. , Vigers, A. J. , Stone, L. , Hellekant, G. , & Kinnamon, S. C. (2005). ATP signaling is crucial for communication from taste buds to gustatory nerves. Science (New York, N.Y.), 310, 1495–1499. 10.1126/science.1118435 16322458

[ejn15799-bib-0019] Friedman, W. J. , Olson, L. , & Persson, H. (1991). Cells that express brain‐derived neurotrophic factor mRNA in the developing postnatal rat brain. The European Journal of Neuroscience, 3, 688–697. 10.1111/j.1460-9568.1991.tb00854.x 12106476

[ejn15799-bib-0020] Fritzsch, B. , Sarai, P. A. , Barbacid, M. , & Silossantiago, I. (1997). Mice with a targeted disruption of the neurotrophin receptor trkB lose their gustatory ganglion cells early but do develop taste buds. International Journal of Developmental Neuroscience, 15, 563–576. 10.1016/S0736-5748(96)00111-6 9263033

[ejn15799-bib-0021] Fundin, B. T. , Silos‐Santiago, I. , Ernfors, P. , Fagan, A. M. , Aldskogius, H. , DeChiara, T. M. , Phillips, H. S. , Barbacid, M. , et al. (1997). Differential dependency of cutaneous mechanoreceptors on neurotrophins, TrK receptors, and P75 LNGFR. Developmental Biology, 190, 94–116. 10.1006/dbio.1997.8658 9331334

[ejn15799-bib-0022] Gailani, M. R. , & Bale, A. E. (1997). Developmental genes and cancer: Role of patched in basal cell carcinoma of the skin. Journal of the National Cancer Institute, 89, 1103–1109. 10.1093/jnci/89.15.1103 9262247

[ejn15799-bib-0023] Gerdes, J. , Schwab, U. , Lemke, H. , & Stein, H. (1983). Production of a mouse monoclonal antibody reactive with a human nuclear antigen associated with cell proliferation. International Journal of Cancer, 31, 13–20. 10.1002/ijc.2910310104 6339421

[ejn15799-bib-0024] Gonzales, C. , Zaleska, M. M. , Riddell, D. R. , Atchison, K. P. , Robshaw, A. , Zhou, H. , & Sukoff Rizzo, S. J. (2014). Alternative method of oral administration by peanut butter pellet formulation results in target engagement of BACE1 and attenuation of gavage‐induced stress responses in mice. Pharmacology, Biochemistry, and Behavior, 126, 28–35. 10.1016/j.pbb.2014.08.010 25242810

[ejn15799-bib-0025] Gonzalez, A. R. , Etchichury, D. , Gil, M. E. , & Del Aguila, R. (2019). Neoadjuvant vismodegib and Mohs micrographic surgery for locally advanced periocular basal cell carcinoma. Ophthalmic Plastic & Reconstructive Surgery, 35, 56–61. 10.1097/IOP.0000000000001166 30444747

[ejn15799-bib-0026] Hirota, M. , Ito, T. , Okudela, K. , Kawabe, R. , Hayashi, H. , Yazawa, T. , Fujita, K. , & Kitamura, H. (2001). Expression of cyclin‐dependent kinase inhibitors in taste buds of mouse and hamster. Tissue & Cell, 33, 25–32. 10.1054/tice.2000.0146 11292167

[ejn15799-bib-0027] Huang, Y. A. , Stone, L. M. , Pereira, E. , Yang, R. , Kinnamon, J. C. , Dvoryanchikov, G. , Chaudhari, N. , Finger, T. E. , Kinnamon, S. C. , & Roper, S. D. (2011). Knocking out P2X receptors reduces transmitter secretion in taste buds. The Journal of Neuroscience : The Official Journal of the Society for Neuroscience, 31, 13654–13661. 10.1523/JNEUROSCI.3356-11.2011 21940456 PMC3188419

[ejn15799-bib-0028] Ito, A. , & Nosrat, C. A. (2009). Gustatory papillae and taste bud development and maintenance in the absence of TrkB ligands BDNF and NT‐4. Cell and Tissue Research, 337, 349–359. 10.1007/s00441-009-0833-7 19629530

[ejn15799-bib-0029] Keefe, K. M. , Sheikh, I. S. , & Smith, G. M. (2017). Targeting neurotrophins to specific populations of neurons: NGF, BDNF, and NT‐3 and their relevance for treatment of spinal cord injury. International Journal of Molecular Sciences, 18, 548. 10.3390/ijms18030548 28273811 PMC5372564

[ejn15799-bib-0030] Knapp, L. , Lawton, A. , Oakley, B. , Wong, L. , & Zhang, C. (1995). Keratins as markers of differentiated taste cells of the rat. Differentiation; Research in Biological Diversity, 58, 341–349. 10.1046/j.1432-0436.1995.5850341.x 7542613

[ejn15799-bib-0031] Kondo, Y. , Murayama, Y. , Konishi, H. , Morimura, R. , Komatsu, S. , Shiozaki, A. , Kuriu, Y. , Ikoma, H. , Kubota, T. , Nakanishi, M. , Ichikawa, D. , Fujiwara, H. , Okamoto, K. , Sakakura, C. , Takahashi, K. , Inoue, K. , Nakajima, M. , & Otsuji, E. (2014). Fluorescent detection of peritoneal metastasis in human colorectal cancer using 5‐aminolevulinic acid. International Journal of Oncology, 45, 41–46. 10.3892/ijo.2014.2417 24821500 PMC4079156

[ejn15799-bib-0032] Lee, E. , & Son, H. (2009). Adult hippocampal neurogenesis and related neurotrophic factors. BMB Reports, 42, 239–244. 10.5483/BMBRep.2009.42.5.239 19470236

[ejn15799-bib-0033] Lindholm, D. , Hamner, S. , & Zirrgiebel, U. (1997). Neurotrophins and cerebellar development. Perspectives on Developmental Neurobiology, 5, 83–94. 9509520

[ejn15799-bib-0034] Liu, H. X. , Ermilov, A. , Grachtchouk, M. , Li, L. , Gumucio, D. L. , Dlugosz, A. A. , & Mistretta, C. M. (2013). Multiple Shh signaling centers participate in fungiform papilla and taste bud formation and maintenance. Developmental Biology, 382, 82–97. 10.1016/j.ydbio.2013.07.022 23916850 PMC3968530

[ejn15799-bib-0035] Lu, W. J. , Mann, R. K. , Nguyen, A. , Bi, T. , Silverstein, M. , Tang, J. Y. , Chen, X. , & Beachy, P. A. (2018). Neuronal delivery of hedgehog directs spatial patterning of taste organ regeneration. Proceedings of the National Academy of Sciences of the United States of America, 115, E200–e209. 10.1073/pnas.1719109115 29279401 PMC5777079

[ejn15799-bib-0036] McLaughlin, S. K. , McKinnon, P. J. , & Margolskee, R. F. (1992). Gustducin is a taste‐cell‐specific G protein closely related to the transducins. Nature, 357, 563–569. 10.1038/357563a0 1608467

[ejn15799-bib-0037] Meng, L. , Ohman‐Gault, L. , Ma, L. , & Krimm, R. F. (2015). Taste bud‐derived BDNF is required to maintain normal amounts of innervation to adult taste buds. eNeuro, 2, ENEURO.0097. 10.1523/ENEURO.0097-15.2015 26730405 PMC4697083

[ejn15799-bib-0038] Mistretta, C. M. , & Kumari, A. (2019). Hedgehog signaling regulates taste organs and oral sensation: Distinctive roles in the epithelium, stroma, and innervation. International Journal of Molecular Sciences, 20, 1341. 10.3390/ijms20061341 30884865 PMC6471208

[ejn15799-bib-0039] Miura, H. , Kusakabe, Y. , & Harada, S. (2006). Cell lineage and differentiation in taste buds. Archives of Histology and Cytology, 69, 209–225. 10.1679/aohc.69.209 17287576

[ejn15799-bib-0040] Miura, H. , Kusakabe, Y. , Sugiyama, C. , Kawamatsu, M. , Ninomiya, Y. , Motoyama, J. , & Hino, A. (2001). Shh and Ptc are associated with taste bud maintenance in the adult mouse. Mechanisms of Development, 106, 143–145. 10.1016/S0925-4773(01)00414-2 11472844

[ejn15799-bib-0041] Miura, H. , Scott, J. K. , Harada, S. , & Barlow, L. A. (2014). Sonic hedgehog‐expressing basal cells are general post‐mitotic precursors of functional taste receptor cells. Developmental Dynamics: An Official Publication of the American Association of Anatomists, 243, 1286–1297. 10.1002/dvdy.24121 24590958 PMC4152395

[ejn15799-bib-0042] Nelson, G. , Hoon, M. A. , Chandrashekar, J. , Zhang, Y. , Ryba, N. J. , & Zuker, C. S. (2001). Mammalian sweet taste receptors. Cell, 106, 381–390. 10.1016/S0092-8674(01)00451-2 11509186

[ejn15799-bib-0043] Nelson, G. M. , & Finger, T. E. (1993). Immunolocalization of different forms of neural cell adhesion molecule (NCAM) in rat taste buds. Journal of Comparative Neurology, 336, 507–516. 10.1002/cne.903360404 8245223

[ejn15799-bib-0044] Nosrat, C. A. , Blomlöf, J. , Elshamy, W. M. , Ernfors, P. , & Olson, L. (1997). Lingual deficits in BDNF and NT3 mutant mice leading to gustatory and somatosensory disturbances, respectively. Development, 124, 1333–1342. 10.1242/dev.124.7.1333 9118804

[ejn15799-bib-0045] Nosrat, I. V. , Agerman, K. , Marinescu, A. , Ernfors, P. , & Nosrat, C. A. (2004). Lingual deficits in neurotrophin double knockout mice. Journal of Neurocytology, 33, 607–615. 10.1007/s11068-005-3330-2 16217617

[ejn15799-bib-0046] Nosrat, I. V. , Lindskog, S. , Seiger, Å. , & Nosrat, C. A. (2000). Lingual BDNF and NT‐3 mRNA expression patterns and their relation to innervation in the human tongue: Similarities and differences compared with rodents. The Journal of Comparative Neurology, 417, 133–152. 10.1002/(SICI)1096-9861(20000207)417:2<133::AID-CNE1>3.0.CO;2-I 10660893

[ejn15799-bib-0047] Nosrat, I. V. , Margolskee, R. F. , & Nosrat, C. A. (2012). Targeted taste cell‐specific overexpression of brain‐derived neurotrophic factor in adult taste buds elevates phosphorylated TrkB protein levels in taste cells, increases taste bud size, and promotes gustatory innervation. The Journal of Biological Chemistry, 287, 16791–16800. 10.1074/jbc.M111.328476 22442142 PMC3351349

[ejn15799-bib-0048] Okubo, T. , Clark, C. , & Hogan, B. L. (2009). Cell lineage mapping of taste bud cells and keratinocytes in the mouse tongue and soft palate. Stem Cells (Dayton, Ohio), 27, 442–450. 10.1634/stemcells.2008-0611 19038788 PMC4337989

[ejn15799-bib-0049] Schroer, A. B. , Branyan, K. W. , Gross, J. D. , Chantler, P. D. , Kimple, A. J. , Vandenbeuch, A. , & Siderovski, D. P. (2021). The stability of tastant detection by mouse lingual chemosensory tissue requires regulator of G protein signaling‐21 (RGS21). Chemical Senses, 46, bjab048. 10.1093/chemse/bjab048 34718440 PMC8785950

[ejn15799-bib-0050] Sekulic, A. , Migden, M. R. , Oro, A. E. , Dirix, L. , Lewis, K. D. , Hainsworth, J. D. , Solomon, J. A. , Yoo, S. , Arron, S. T. , Friedlander, P. A. , Marmur, E. , Rudin, C. M. , Chang, A. L. S. , Low, J. A. , Mackey, H. M. , Yauch, R. L. , Graham, R. A. , Reddy, J. C. , & Hauschild, A. (2012). Efficacy and safety of vismodegib in advanced basal‐cell carcinoma. The New England Journal of Medicine, 366, 2171–2179. 10.1056/NEJMoa1113713 22670903 PMC5278761

[ejn15799-bib-0051] Sohn, W. J. , Gwon, G. J. , An, C. H. , Moon, C. , Bae, Y. C. , Yamamoto, H. , Lee, S. , & Kim, J. Y. (2011). Morphological evidences in circumvallate papilla and von Ebners' gland development in mice. Anat Cell Biol, 44, 274–283. 10.5115/acb.2011.44.4.274 22254156 PMC3254881

[ejn15799-bib-0052] Sternini, C. (2013). In search of a role for carbonation: Is this a good or bad taste? Gastroenterology, 145, 500–503. 10.1053/j.gastro.2013.07.018 23891610 PMC4416054

[ejn15799-bib-0053] Takeda, M. , Suzuki, Y. , Obara, N. , & Nagai, Y. (1992). Neural cell adhesion molecule of taste buds. Journal of Electron Microscopy, 41, 375–380. 1487689

[ejn15799-bib-0054] Vutskits, L. , Djebbara‐Hannas, Z. , Zhang, H. , Paccaud, J. P. , Durbec, P. , Rougon, G. , Muller, D. , & Kiss, J. Z. (2001). PSA‐NCAM modulates BDNF‐dependent survival and differentiation of cortical neurons. The European Journal of Neuroscience, 13, 1391–1402. 10.1046/j.0953-816x.2001.01516.x 11298800

[ejn15799-bib-0055] Yang, H. , Cong, W. N. , Yoon, J. S. , & Egan, J. M. (2014). Vismodegib, an antagonist of hedgehog signaling, directly alters taste molecular signaling in taste buds. Cancer Medicine, 4, 245–252. 10.1002/cam4.350 25354792 PMC4329008

[ejn15799-bib-0056] Yang, T. , Kersigo, J. , Jahan, I. , Pan, N. , & Fritzsch, B. (2011). The molecular basis of making spiral ganglion neurons and connecting them to hair cells of the organ of Corti. Hearing Research, 278, 21–33. 10.1016/j.heares.2011.03.002 21414397 PMC3130837

[ejn15799-bib-0057] Yao, P. J. , Petralia, R. S. , & Mattson, M. P. (2016). Sonic hedgehog signaling and hippocampal neuroplasticity. Trends in Neurosciences, 39, 840–850. 10.1016/j.tins.2016.10.001 27865563 PMC5148655

[ejn15799-bib-0058] Yates, J. M. , Smith, K. G. , & Robinson, P. P. (2004). The effect of brain‐derived neurotrophic factor on sensory and autonomic function after lingual nerve repair. Experimental Neurology, 190, 495–505. 10.1016/j.expneurol.2004.08.010 15530888

[ejn15799-bib-0059] Zhang, C. , Cotter, M. , Lawton, A. , Oakley, B. , Wong, L. , & Zeng, Q. (1995). Keratin 18 is associated with a subset of older taste cells in the rat. Differentiation; Research in Biological Diversity, 59, 155–162. 10.1046/j.1432-0436.1995.5930155.x 7589899

